# 吉西他滨单药/或联合铂类与紫杉类单药/或联合铂类不同顺序治疗晚期肺鳞癌的疗效与安全性

**DOI:** 10.3779/j.issn.1009-3419.2015.05.09

**Published:** 2015-05-20

**Authors:** 晶 许, 晓晴 刘, 红军 高, 万峰 郭, 传昊 汤, 晓燕 李, 俭杰 李, 海峰 秦, 伟霞 王, 莉莉 曲, 红 王, 辉 杨, 琳 杨

**Affiliations:** 100071 北京，解放军第307医院肺部肿瘤内科 Department of Lung Cancer, Affiliated Hospital of Academy of Military Medical Sciences, Beijing 100071, China

**Keywords:** 肺肿瘤, 化疗, 顺序治疗, Lung neoplasms, Chemotherapy, Sequential treatment

## Abstract

**背景与目的:**

吉西他滨和紫杉类药物是晚期肺鳞癌常用的化疗药物，但二者在一、二线治疗中的最佳顺序尚未明确，本研究旨在分析吉西他滨单药/或联合铂类同紫杉类药物单药/或联合铂类在一、二线治疗中不同化疗顺序的疗效及毒副作用。

**方法:**

回顾性分析了105例Ⅲb期-Ⅳ期肺鳞癌患者，49例为一线予吉西他滨单药/或联合铂类，进展后二线予紫杉类单药/或联合铂类治疗（G-T组）；56例为一线予紫杉类单药/或联合铂类，进展后二线予吉西他滨单药/或联合铂类治疗（T-G组）。主要研究终点为总生存（overall survival, OS），次要研究终点为无进展生存期（progression-free survival, PFS）、客观缓解率（objective response rate, ORR）、疾病控制率（disease control rate, DCR）及不良反应。

**结果:**

① G-T组与T-G组中位OS分别为为18.5个月和19.0个月（*P*=0.520）。②G-T组与T-G组一线化疗中位PFS1分别为5.0个月和4.0个月（*P*=0.584）；两组二线化疗中位PFS2为2.5个月和2.7个月（*P*=0.432）。③G-T组与T-G组一线化疗ORR1分别为36.73%和33.92%（*P*=0.577），DCR1分别为79.59%和89.29%（*P*=0.186）；二线化疗ORR2分别为4.08%和5.36%（*P*=0.085），DCR2分别为51.02%和66.07%（*P*=0.118）。④G-T组与T-G组毒副作用相似，但G-T组更易出现Ⅲ级-Ⅳ级红细胞减低（*P*=0.027）和血小板减低（*P*=0.002）。

**结论:**

一线吉西他滨单药/或联合铂类序贯二线紫杉类单药/或联合铂类与一线紫杉类单药/或联合铂类序贯二线吉西他滨单药/或联合铂类疗效相当，不良反应可耐受，两种序贯模式均对初治晚期肺鳞癌患者有效。

## Abstract (en)

**Background and objective:**

Gemcitabine and taxanes are effective agents commonly used in advanced squamous lung cancer. The best treatment sequence, however, is unclear to our knowledge. So we conducted this retrospective study in order to compare the efficacy and toxicities of first-line Gemcitabine +/- platinum followed by second-line taxanes +/- platinum with the reverse sequence.

**Methods:**

We totally analyzed 105 patients with stage Ⅲb-Ⅳ squamous lung cancer in our retrospective study. There were 49 patients receiving gemcitabine +/- platinum first-line followed by taxanes +/- platinum second-line (G-T group), and 56 patients receiving taxanes +/- platinum first-line followed by gemcitabine +/- platinum second-line (T-G group). The primary endpoint of the study was overall survival (OS), and the secondary endpoints included progression-free survival (PFS), objective response rate (ORR), disease control rate (DCR) and toxicities.

**Results:**

The median OS were 18.5 mo in G-T group and 19.0 mo in T-G group (*P*=0.520). The median PFS1 was 5.0 mo and 4.0 mo with first-line gemcitabine +/- platinum and taxanes +/- platinum, respectively (*P*=0.584). The median PFS2 was 2.7 mo and 2.5 mo with second-line gemcitabine +/- platinum and taxanes +/- platinum (*P*=0.432). The ORR1 of G-T group and T-G group were 36.73% and 33.92% (*P*=0.577), and DCR1 were 79.59% and 89.29% (*P*=0.186); the ORR2 of G-T group and T-G group were 4.08% and 5.36% (*P*=0.085), and DCR2 were 51.02% and 66.07%, respectively (*P*=0.118). Hematologic toxicities was more frequent in G-T group, the patients experienced more grade 3-4 lower hemoglobin (*P*=0.027) and thrombocytopenia (*P*=0.002).

**Conclusion:**

The efficacy of first line gemcitabine +/- platinum followed by second line taxanes +/- platinum and the reverse sequence was similar, and the toxicities was tolerable. Both sequential patterns were effective in advanced squamous lung cancer.

在全世界范围内，肺癌的发病率和死亡率均居榜首^[1]^。在非小细胞肺癌（non-small cell lung cancer, NSCLC）中，肺鳞癌的发病率约占30%。由于症状、体征不具特异性，并且缺乏有效的早期诊断手段，约75%的NSCLC一经诊断即为Ⅲb期-Ⅳ期，对于这些失去手术机会的患者，细胞毒性药物化疗为当前的主要治疗方法^[2, 3]^。

目前，第三代化疗药物联合铂类为NSCLC的一线标准化疗方案^[4-7]^，多项随机临床试验^[8-12]^均证明新一代化疗药物疗效相似，且对于晚期肺鳞癌而言，一线使用吉西他滨较培美曲塞而言有更好的疗效和生存获益^[13]^。然而，一线化疗不可避免产生耐药，大多数患者会进入二线化疗。多西他赛及培美曲塞为当前推荐的二线化疗药物^[14-17]^，对于晚期肺鳞癌患者而言，二线选择多西他赛较培美曲塞则更能获益^[16, 17]^。

在临床实践中，医生常根据患者的组织学类型、美国东部肿瘤协作组体力评分（Eastern Cooperative Oncology Group Performance Status, ECOG PS）、经济状况、既往治疗、耐受程度等来选择合适的一线、二线化疗药物。吉西他滨和紫杉类药物常常用来作为晚期肺鳞癌一线和/或二线化疗药物。

近年来，序贯治疗是肿瘤研究中的热点话题之一，其在晚期肺鳞癌细胞毒性化疗中缺乏相应的研究，在临床实践中，晚期肺鳞癌一线、二线最佳药物的治疗顺序至今仍缺乏相应数据支持。为了研究化疗顺序对疗效及安全性的影响，本项回顾性研究主要对比了一线吉西他滨单药/或联合铂类序贯二线紫杉类药物单药/或联合铂类与一线紫杉类药物单药/或联合铂类序贯二线吉西他滨单药/或联合铂类治疗晚期肺鳞癌的疗效与安全性，以期探索最佳治疗模式。

## 1 资料和方法

### 1.1 病例与分组

选取2008年1月-2014年8月就诊于解放军第307医院肺部肿瘤内科的晚期肺鳞癌患者105例。其病理类型全部经组织学和/或细胞学检查证实。均为Ⅲb期或Ⅳ期。其病灶为可测量、可评估病灶。所有患者化疗前从未接受过任何抗肿瘤治疗，ECOG PS评分0分-2分，其基线血常规、肝肾功、出凝血功能、尿便常规、心电图检查均符合化疗适应证。

105例晚期肺鳞癌患者被分为两组：一线吉西他滨单药/或联合铂类进展后二线紫杉类药物单药/或联合铂类组（G-T组）49例，一线紫杉类药物单药/或联合铂类进展后二线吉西他滨单药/或联合铂类（T-G组）56例。

### 1.2 治疗方案

吉西他滨1, 000 mg/m^2^-1, 250 mg/m^2^，静脉滴注（30 min内）d1，d8；多西他赛60 mg/m^2^-75 mg/m^2^，静滴，d1；紫杉醇150 mg/m^2^-175 mg/m^2^，静滴，d1。若联合顺铂：顺铂75 mg/m^2^，静滴，d1（或分3天给药）；若联合卡铂：卡铂曲线下面积（area under the curve, AUC）=5-6，静滴，d1（或分3天给药）。

化疗前常规预防呕吐，化疗后出现的呕吐、血象异常及肝肾功异常等不良反应均按常规处理。本研究允许由于毒性引起的药物中断或剂量调整。

疗效评估：2个周期化疗结束后在1周之内行胸部计算机断层扫描（computed tomography, CT）、浅表淋巴结超声、腹部超声等评效，按照实体瘤疗效评价标准（Response Evaluation Criteria in Solid Tumors, RECIST）进行疗效评价。

不良反应评价按抗肿瘤药物不良反应的分度标准（世界卫生组织标准）分为Ⅰ度-Ⅳ度。

### 1.3 研究终点

本研究主要研究终点为：患者的总生存（overall survival, OS），次要研究终点为：无进展生存（progression-free survival, PFS）、客观缓解率（objective response rate, ORR）、疾病控制率（disease control rate, DCR）及不良反应分析。

本研究中OS为患者从一线化疗开始到任何原因引起患者死亡的时间；PFS1为患者从一线化疗开始到一线治疗疾病发生进展的时间，PFS2为患者从二线化疗开始到二线治疗疾病发生进展或死亡的时间；ORR1为经过一线治疗CR+PR的病例总数占对于可评价的总体病例数的比例，ORR2为经过二线治疗CR+PR的病例总数占对于可评价的总体病例数的比例；DCR1为经过一线治疗CR+PR+SD的病例总数占对于可评价的总体病例数的比例，DCR2为二线治疗CR+PR+SD的病例总数占对于可评价的总体病例数的比例。

### 1.4 统计学方法

采用IBM SPSS statistics（version 19）软件对数据进行分析，定性资料采用R×C表的*χ*^2^检验或*Fisher*确切概率法检验，定量资料采用两样本比较的t检验或非参数秩和检验，生存分析采用*Kaplan*-*Meier*曲线，生存比较采用*Log*-*rank*检验，以*P* < 0.05为差异有统计学意义。

## 2 结果

### 2.1 入组患者基线临床特征和伴随治疗情况

筛选的105例鳞癌患者均按上述入组及排除标准纳入，两组患者临床特征及治疗情况未见统计学差异（表 1）。

**1:** 两组患者一般资料的比较 The base-line characteristics of the groups

Characteristics	G-T group (*n*=49)	T-G group (*n*=56)	*P*
Gender			0.740
Male	39 (79.6%)	46 (82.1%)	
Female	10 (20.4%)	10 (17.9%)	
Age (yr)			0.647
Median (range)	62 (39-78)	59 (37-83)	
Smoking status			0.521
Ever-smoker	34 (69.4%)	42 (75.0%)	
Never-smoker	15 (30.6%)	14 (25.0%)	
Disease stage			0.425
Ⅲb	6 (12.2%)	10 (17.9%)	
Ⅳ	43 (87.8%)	46 (82.1%)	
ECOG PS			0.206
0	0 (0)	2 (3.6%)	
1	46 (93.9%)	47 (83.9%)	
2	3 (6.1%)	7 (12.5%)	
Thoracic radiotherapy			0.353
Performed	15 (30.6%)	22 (39.3%)	
Not performed	34 (69.4%)	34 (60.7%)	
Combined with platinum			0.554
G (T)+platinum-T (G)+platinuem	28 (57.1%)	36 (64.3%)	
G (T)+platinum-G (T)	16 (32.7%)	13 (23.2%)	
G (T)-T (G)	5 (10.2%)	7 (12.5%)	
First-line platinum drugs			0.440
Carboplatin	16 (32.7%)	24 (42.9%)	
Cisplatin	28 (57.1%)	25 (44.6%)	
Single agent	5 (10.2%)	7 (12.5%)	
Second-line platinum drugs			0.648
Carboplatin	19 (38.8%)	22 (39.3%)	
Cisplatin	9 (18.4%)	14 (25.0%)	
Single agent	21 (42.9%)	20 (35.7%)	
Taxanes drugs			0.582
Docetaxel	28 (57.1%)	29 (51.8%)	
Paclitaxel	21 (42.9%)	27 (48.2%)	
G-T group: patients receiving gemcitabine +/- platinum first-line followed by taxanes +/- platinum second-line; T-G group: patients receiving taxanes +/- platinum first-line followed by gemcitabine +/- platinum second-line. ECOG PS: Eastern Cooperative Oncology Group Performance Status.			

### 2.2 疗效分析

#### 2.2.1 OS

截止到2014年8月1日，105例患者中通过查阅病历，门诊、住院随访及电话随访共有61例确定已死亡，21例失访，23例仍在随访中。G-T组中位OS为18.5个月，T-G组中位OS为19.0个月，二组OS未见统计学差异（*P*=0.520），生存曲线见图 1A。

**1:**
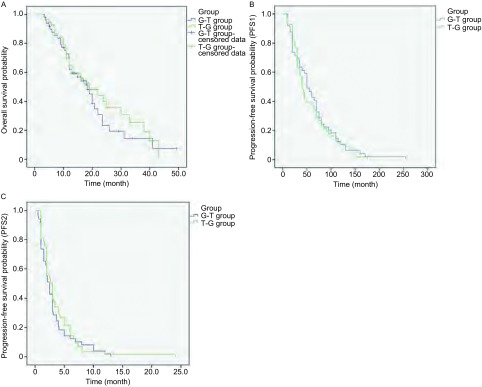
G-T组和G-T组生存曲线。A：G-T组和T-G组OS比较；B：G-T组和T-G组一线化疗PFS（PFS1）比较；C：G-T组和T-G组二线化疗PFS（PFS2）比较 *Kaplan*-*Meier* curves of survival in G-T group and T-G group. A: *Kaplan*-*Meier* curves of OS in G-T group and T-G group; B: *Kaplan*-*Meier* curves of first-line PFS1 in G-T group and T-G group; C: *Kaplan*-*Meier* curves of second-line PFS2 in G-T group and T-G group

#### 2.2.2 PFS

一线化疗PFS1：G-T组中位PFS1为5.0个月，T-G组中位PFS1为4.0个月，PFS1未见明显统计学差异（*P*=0.584)，生存曲线见图 1B。

二线化疗PFS2：T-G组中位PFS2为2.7个月，G-T组PFS2为2.5个月，二者相比未见明显统计学差异（*P*=0.432），生存曲线见图 1C。

#### 2.2.3 近期疗效

一线化疗ORR1：G-T组36.73%（18/49）和T-G组33.92%（19/56）（*P*=0.577）；二线化疗ORR2：T-G组5.36%（3/56）和G-T组4.08%（2/49）（*P*=0.085）。一线化疗DCR1：G-T组79.59%（39/49）和T-G组89.29%（50/56）（*P*=0.186）；二线化疗DCR2：T-G组66.07%（37/56）和G-T组51.02%（25/49）（*P*=0.118）（表 2）。

**2:** 两组生存情况与客观缓解率比较 Comparison of survival and objective response between two groups

Item	G-T group (*n*=49)	T-G group (*n*=56)	*P*
First-line PFS (PFS1)	5.0 (3.71-6.27)	4.0 (3.35-4.65)	0.584
Second-line PFS (PFS2)	2.5 (1.93-3.07)	2.7 (1.89-3.52)	0.432
OS	18.5 (14.34-22.66)	19.0 (10.39-27.62)	0.520
1-year survival	60.2%	63.4 %	0.729
2-year survival	23.0%	40.2%	0.425
First-line ORR (ORR1)	36.73%	33.92%	0.577
Second-line ORR (ORR2)	4.08%	5.36%	0.085
First-line DCR (DCR1)	79.59%	89.29%	0.186
Second-line DCR (DCR2)	51.02%	66.07%	0.118
PFS: progression free survival; OS: overall survival; ORR: objective response rate; DCR: disease control rate.			

### 2.3 不良反应分析

研究主要比较了两组方案的血液学毒性及非血液学毒性（消化道反应、肝功损害、肾功损害），除红细胞减少及血小板减少两组具有统计学差异外，其余毒性相当（表 3）。

**3:** 两组患者不良反应比较 Comparison of toxicities between two groups

Toxicities	G-T group (*n*=49)					T-G group (*n*=56)				P^*^	*P*↑
	Ⅰ	Ⅱ	Ⅲ	Ⅳ		Ⅰ	Ⅱ	Ⅲ	Ⅳ		
Leukopenia	3 (6.12%)	8 (16.33%)	4 (8.16%)	17 (34.69%)		8 (14.29%)	8 (14.29%)	7 (12.50%)	10 (17.86%)	0.252	0.184
Granulocytopenia	4 (8.16%)	12 (24.49%)	7 (14.29%)	9 (18.37%)		4 (7.14%)	6 (10.71%)	5 (8.93%)	9 (16.07%)	0.166	0.386
Lower hemoglobin	5 (10.20%)	9 (18.37%)	4 (8.16%)	13 (26.53%)		14 (25.00%)	13 (23.21%)	5 (8.93%)	4 (7.14%)	0.049	0.027
Thrombocytopenia	2 (4.08%)	2 (4.08%)	3 (6.12%)	13 (26.53%)		3 (5.36%)	3 (5.36%)	3 (5.36%)	2 (3.57%)	0.021	0.002
Gastrointestinal reaction	10 (20.41%)	10 (20.41%)	2 (4.08%)	0 (0)		9 (16.07%)	8 (14.29%)	0 (0)	1 (1.79%)	0.321	0.597
Renal dysfunction	16 (32.65%)	2 (4.08%)	0 (0)	0 (0)		14 (25.00%)	5 (8.93%)	0 (0)	0 (0)	0.475	—
Hepatic dysfunction	13 (26.53%)	2 (4.08%)	0 (0)	0 (0)		6 (10.71%)	3 (5.36%)	0 (0)	0 (0)	0.110	—
P^*^: Test including all grades; *P*↑: test comparing grade >Ⅲ and grade < Ⅱ.											

## 3 讨论

近年来，在NSCLC中有关治疗顺序的研究主要集中于靶向药物与化疗药物上，由于不同药物的作用机制与耐药机理不尽相同，一种药物可能会影响另一种药物发挥作用的信号通路，故不同治疗顺序可能会带来的不同的疗效与毒性。Giovannetti等^[18, 19]^发现，表皮生长因子受体酪氨酸激酶抑制剂（epidermal growth factor receptor-tyrosine kinase inhibitors, EGFR-TKIs）会抑制与培美曲塞耐药相关的酶胸苷酸合成酶（thymidylate synthase, TYMS），而培美曲塞则会诱导与EGFR-TKIs耐药相关蛋白AKT的磷酸化^[20]^，从而导致二者不同治疗顺序疗效的差异^[21]^。目前，对晚期肺鳞癌化疗顺序的研究相对缺乏，而紫杉醇类药物和吉西他滨是晚期肺鳞癌有效的化疗药物，且二者有不同的作用机制与耐药机理，这两个药物的治疗顺序是否对生存及安全性有不同的作用和影响，为了明确这个问题，本研究将105例Ⅲb期-Ⅳ期肺鳞癌患者分为两组：一线吉西他滨单药/或联合铂类进展后二线予紫杉类单药/或联合铂类化疗，一线紫杉类单药/或联合铂类进展后二线予吉西他滨单药/或联合铂类化疗，旨在观察这两种化疗顺序对疗效及安全性的潜在影响。

为观察治疗顺序对疗效影响，本研究尽可能保证了治疗前两组患者性别、年龄、吸烟史、ECOG PS评分、分期等临床资料的均衡，并且确保患者除治疗顺序不同外，其他治疗情况（紫杉类药物使用情况、放疗情况、铂类联合情况、化疗剂量）的均衡。

本研究PFS1与PFS2、ORR1与ORR2、DCR1与DCR2结果均与大部分临床试验数据接近，但由于本研究主要目的是比较治疗顺序对疗效的影响，纳入的患者治疗情况较为复杂，故很难与某一个具体的临床试验相比较。值得一提的是，两组中位OS分别为18.5个月与19.0个月，1年生存率G-T组为60.2%，T-G组为63.4%，2年生存率G-T组为23.0%，T-G组为40.2%。但是据ECOG1594报道^[9]^，一线使用吉西他滨、紫杉醇、多西他赛联合顺铂的中位OS近8个月，1年、2年生存率分别为30%左右及10%左右，TAX326^[8]^紫杉醇联合顺铂/卡铂中位OS也只有11.3个月，1年、2年生存率分别为46%和21%，JMDB研究^[13]^鳞癌亚组分析也显示吉西他滨联合顺铂中位OS为10.8个月，而本研究OS较上述延长了近9个月，1年生存率及2年生存率也明显较高，其原因可能是本研究35%（37/105）的患者在研究过程中接受了放疗的干预，并且约62%（65/105）的患者在一线二线之后又接受了三线以上的化疗、靶向治疗或放疗、粒子植入、手术治疗。另外，本研究T-G组的2年生存率较G-T组有增高的趋势，而在后续治疗上，除靶向治疗（EGFR-TKIs）G-T组较T-G组人数多外（*P*=0.027）其余的后续治疗两组并无统计学差异（*P*=0.664）。由此推测在后续治疗中，G-T组接受靶向治疗的患者可能并未从中获益，亦或是G-T组接受靶向治疗的患者本身ECOG PS评分就较低，医生更倾向于给予其靶向治疗，这两个原因均有可能使G-T组2年生存率较低，但仍需要后续研究进一步证实。

在毒性方面，两组血液学毒性在白细胞减少、粒细胞减少未见统计学差异，而G-T组更易出现Ⅲ级-Ⅳ级红细胞减少、血小板减少（*P* < 0.05）；非血液学毒性二者未见差异。

因为本研究为回顾性分析，样本量有限，且接受治疗的紫杉类药物同时包含紫杉醇和多西他赛，虽然二者抗肿瘤机制相同，但仍具有不同的体内外活性，虽然纳入分析的患者已尽可能确保两组均衡，但仍不可避免的给本研究带来了混杂因素，所以还需要扩大样本量后进一步行亚组分析比较二者的差异。

总之，本研究提示对晚期肺鳞癌而言，一线吉西他滨单药/或联合铂类序贯二线紫杉类单药/或联合铂类与一线紫杉类单药/或联合铂类序贯二线吉西他滨单药/或联合铂类疗效相当，毒副作用尚可耐受。结果初步提示在晚期鳞癌化疗中这两种药物的治疗顺序对疗效影响较小，在临床实践中，可结合患者实际情况，为患者制定最佳的个体化治疗顺序。

## References

[1] Carney DN (2002). Lung cancer--time to move on from chemotherapy. N Engl J Med.

[2] Wu YL, Lu S, Cheng Y (2014). Efficacy and safety of pemetrexed/cisplatin versus gemcitabine/cisplatin as first-line treatment in Chinese patients with advanced nonsquamous non-small cell lung cancer. Lung Cancer.

[3] Chute JP, Chen T, Feigal E (1999). Twenty years of phase Ⅲ trials for patients with extensive-stage small-cell lung cancer: perceptible progress. J Clin Oncol.

[4] Non-small Cell Lung Cancer Collaborative Group (1995). Chemotherapy in non-small cell lung cancer: a *meta*-analysis using updated data on individual patients from 52 randomised clinical trials. BMJ.

[5] NSCLC <italic>Meta</italic>-Analyses Collaborative Group (2008). Chemotherapy in addition to supportive care improves survival in advanced non-small-cell lung cancer: a systematic review and *meta*-analysis of individual patient data from 16 randomized controlled trials. J Clin Oncol.

[6] Souquet PJ, Chauvin F, Boissel JP (1993). Polychemotherapy in advanced non small cell lung cancer: a *meta*-analysis. Lancet.

[7] Azzoli CG, Baker S Jr, Temin S (2009). American Society of Clinical Oncology Clinical Practice Guideline update on chemotherapy for stage Ⅳ non-small-cell lung cancer. J Clin Oncol.

[8] Fossella F, Pereira JR, von Pawel J (2003). Randomized, multinational, phase Ⅲ study of docetaxel plus platinum combinations versus vinorelbine plus cisplatin for advanced non-small-cell lung cancer: the TAX 326 study group. J Clin Oncol.

[9] Fisher MD, D'Orazio A (2000). Phase Ⅱ and Ⅲ trials: comparison of four chemotherapy regimens in advanced non small cell lung cancer (ECOG 1594). Clin Lung Cancer.

[10] Grossi F, Aita M, Defferrari C (2009). Impact of third-generation drugs on the activity of first-line chemotherapy in advanced non-small cell lung cancer: a *meta*-analytical approach. Oncologist.

[11] Scagliotti GV, De Marinis F, Rinaldi M (2002). Phase Ⅲ randomized trial comparing three platinum-based doublets in advanced non-small-cell lung cancer. J Clin Oncol.

[12] Kelly K, Crowley J, Bunn PA Jr (2001). Randomized phase Ⅲ trial of paclitaxel plus carboplatin versus vinorelbine plus cisplatin in the treatment of patients with advanced non--small-cell lung cancer: a Southwest Oncology Group trial. J Clin Oncol.

[13] Scagliotti GV, Parikh P, von Pawel J (2008). Phase Ⅲ study comparing cisplatin plus gemcitabine with cisplatin plus pemetrexed in chemotherapy-naive patients with advanced-stage non-small-cell lung cancer. J Clin Oncol.

[14] Fossella FV, DeVore R, Kerr RN (2000). Randomized phase Ⅲ trial of docetaxel versus vinorelbine or ifosfamide in patients with advanced non-small-cell lung cancer previously treated with platinum-containing chemotherapy regimens. The TAX 320 Non-Small Cell Lung Cancer Study Group. J Clin Oncol.

[15] Shepherd FA, Dancey J, Ramlau R (2000). Prospective randomized trial of docetaxel versus best supportive care in patients with non-small cell lung cancer previously treated with platinum-based chemotherapy. J Clin Oncol.

[16] Hanna N, Shepherd FA, Fossella FV (2004). Randomized phase Ⅲ trial of pemetrexed versus docetaxel in patients with non-small-cell lung cancer previously treated with chemotherapy. J Clin Oncol.

[17] Demarinis F, Paul S, Hanna N (2006). Survival update for the phase Ⅲ study of pemetrexed vs docetaxel in non-small cell lung cancer (NSCLC). J Clin Oncol.

[18] Giovannetti E, Mey V, Nannizzi S (2005). Cellular and pharmacogenetics foundation of synergistic interaction of pemetrexed and gemcitabine in human non-small-cell lung cancer cells. Mol Pharmacol.

[19] Giovannetti E, Lemos C, Tekle C (2008). Molecular mechanisms underlying the synergistic interaction of erlotinib, an epidermal growth factor receptor tyrosine kinase inhibitor, with the multitargeted antifolate pemetrexed in non-small-cell lung cancer cells. Mol Pharmacol.

[20] Janmaat ML, Kruyt FA, Rodriguez JA (2003). Response to epidermal growth factor receptor inhibitors in non-small cell lung cancer cells: limited antiproliferative effects and absence of apoptosis associated with persistent activity of extracellular signal-regulated kinase or Akt kinase pathways. Clin Cancer Res.

[21] Fiala O, Pesek M, Finek J (2013). Sequential treatment of advanced-stage lung adenocarcinoma harboring wild-type *EGFR* gene: second-line pemetrexed followed by third-line erlotinib versus the reverse sequence. Anticancer Res.

